# Reference genes for normalization of qPCR assays in sugarcane plants under water deficit

**DOI:** 10.1186/s13007-017-0178-2

**Published:** 2017-04-17

**Authors:** Larissa Mara de Andrade, Michael dos Santos Brito, Rafael Fávero Peixoto Junior, Paulo Eduardo Ribeiro Marchiori, Paula Macedo Nóbile, Alexandre Palma Boer Martins, Rafael Vasconcelos Ribeiro, Silvana Creste

**Affiliations:** 1 Centro de Cana, Instituto Agronômico (IAC), P.O. Box 206, Ribeirão Preto, SP 14001-970 Brazil; 2 Departamento de Genética, Faculdade de Medicina de Ribeirão Preto, Universidade de São Paulo, Ribeirão Preto, SP 14049-900 Brazil; 3 Centro de Ecofisilogia e Biofísica, IAC, P.O. Box 1481, Campinas, SP 13012-970 Brazil; 4 Departamento de Biologia Vegetal, Instituto de Biologia, Universidade Estadual de Campinas, P.O. Box 6109, Campinas, SP 13083-970 Brazil

**Keywords:** *Saccharum* spp., Water-deprivation, Normalization, NormFinder, RefFinder

## Abstract

**Background:**

Sugarcane (*Saccharum* spp.) is the main raw material for sugar and ethanol production. Among the abiotic stress, drought is the main one that negatively impact sugarcane yield. Although gene expression analysis through quantitative PCR (qPCR) has increased our knowledge about biological processes related to drought, gene network that mediates sugarcane responses to water deficit remains elusive. In such scenario, validation of reference gene is a major requirement for successful analyzes involving qPCR.

**Results:**

In this study, candidate genes were tested for their suitable as reference genes for qPCR analyses in two sugarcane cultivars with varying drought tolerance. Eight candidate reference genes were evaluated in leaves sampled in plants subjected to water deficit in both field and greenhouse conditions. In addition, five genes were evaluated in shoot roots of plants subjected to water deficit by adding PEG8000 to the nutrient solution. NormFinder and RefFinder algorithms were used to identify the most stable gene(s) among genotypes and under different experimental conditions. Both algorithms revealed that in leaf samples, *UBQ1* and *GAPDH* genes were more suitable as reference genes, whereas *GAPDH* was the best reference one in shoot roots.

**Conclusion:**

Reference genes suitable for sugarcane under water deficit were identified, which would lead to a more accurate and reliable analysis of qPCR. Thus, results obtained in this study may guide future research on gene expression in sugarcane under varying water conditions.

**Electronic supplementary material:**

The online version of this article (doi:10.1186/s13007-017-0178-2) contains supplementary material, which is available to authorized users.

## Background

Sugarcane is a monocot with C_4_ metabolism, presenting high photosynthetic efficiency and accumulating sugar, fiber and water in stalk internodes [1]. Worldwide, sugarcane is considered the main raw material for sugar and biofuel production [2]. However, low water availability on sugarcane fields can drastically reduce yield and total recoverable sugar [3, 4]. Despite advances in sugarcane breeding, the lack of knowledge about genetic and molecular responses involved in drought tolerance, and its quantitative heritage, represent the main challenge for the development of tolerant cultivars. Thereby, identification and understanding of genes and signaling networks in sugarcane for overcoming drought conditions are fundamental for the development of new cultivars with enhanced tolerance under water-deprived conditions [5, 6].

Quantitative PCR (qPCR), also known as real time PCR, is an analytical technique that has revolutionized the exploration of gene expression analyses [7]. Among advantages use qPCR are: higher sensibility, real time detection of transcripts, speed of analyses and reproducibility to obtain a gene expression profile [8, 9]. In spite of being an extremely powerful technique for precisely quantifying changes in gene expression, RNA quality and integrity, efficiency of cDNA synthesis, and variations in RNA input amounts can affect qPCR performance and produce no reliable results [7, 8, 10, 11]. To avoid the influence of these factors, a normalization step of gene expression data is essential [9, 11–13] to correct variations present at samples and conditions [13, 14]. To identify suitable reference genes for qPCR analyses, different mathematical algorithms have been proposed, such as NormFinder [11], GeNorm [10], BestKeeper [12], and DeltaCt [15]. RefFinder is another algorithm used for reference gene analysis, grouping all previous algorithms cited above for evaluating a comprehensive ranking of stability genes [16]. Therefore, identification of a suitable reference gene highly and constantly expressed is important in order to obtain reliable results [17].

In literature, several normalization approaches in monocots plants under drought stress conditions have pointed reference genes in different organisms such as rice [14], maize [18], wheat [19], sorghum [20], wheat [21], and sugarcane [22–24]. However, plants under drought stress revealed that reference genes exhibit stability variations of gene expression according to genotype, tissue, phenological stage and experimental conditions [13, 14]. Thus, the objective of this study was to evaluate the candidate reference genes stability in two sugarcane genotypes under water deficit conditions. Therefore, we applied NormFinder and RefFinder free statistical algorithms to evaluate the expression stability of several candidate reference genes on a set of experiments imposing water deficit to sugarcane in different ways. Our findings revealed the most suitable genes for using as reference in qPCR assays focused on RNA transcripts quantification of sugarcane under water deficit.

## Methods

### Plant materials and experimental conditions

Two sugarcane (*Saccharum* spp.) genotypes developed by the “Programa Cana” (Instituto Agronômico, Brazil) were studied: ‘IACSP94-2094’ and ‘IACSP97-7065’. These genotypes have differential growth and yield in drought-prone areas of Brazilian Cerrado, with ‘IACSP94-2094’ being more drought tolerant than ‘IACSP97-7065’ [25]. Both genotypes were analyzed in three independent experiments: on field, in greenhouse conditions and in greenhouse under hydroponic conditions; in all of them, both genotypes underwent well-watered (control) and drought stressed.

The field trial was carried out in Goianésia, GO, Brazil (15°13′S; 48°56′W) during the dry season, from April to September. Leaf samples (leaf +1) of first-cut plants were collected between 9:00 and 9:30 a.m. in irrigated (the irrigation was applied by linear sprinkler system) and non-irrigated areas during experiment: 42, 89, and 117 days after the last rainfall, when plants were six, seven and nine months old respectively.

The greenhouse trial was carried out in Campinas SP, Brazil (22°52′S; 47°44′W), and both genotypes were grown in the same tanks (0.6 m^3^) containing soil previously fertilized according to Van Raij et al. [26]. Leaf samples (leaf +1) from six months plants were collected between 9:00 and 9:30 a.m. in irrigated and non-irrigated treatments at three times: 15 and 21 days after water withholding deficit and also after nine days of soil rehydration for evaluating plant recovery. For more details about field and greenhouse trials, see Andrade et al. [25].

**Fig. 1:**
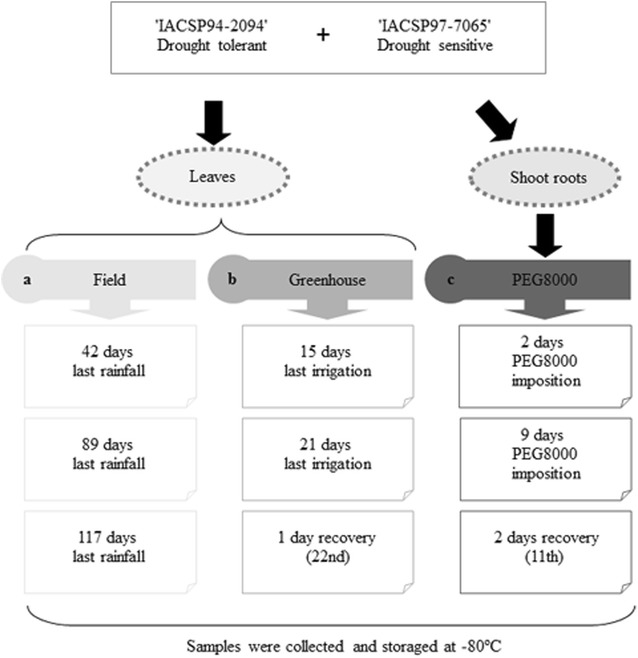
Schematic diagram of the experiments used in reference genes selection. **a** Experiment conduced on field. **b** Experiment conduced at greenhouse. **c** Experiment conduced at greenhouse using PEG8000 treatment. *Each box* means the moment of sampling. The *numbers* inside the bracts mean the number of days under treatment

The hydroponic trial was conducted in greenhouse at the ‘Santa Elisa’ farm, Campinas, SP, Brazil (22°52′S; 47°44′W). The plants were cultivated in plastic boxes (12L) containing nutritive solution (osmotic potential of −0.11 MPa) composed by (in mmol L^−1^) 15 de N (7% as NH_4_^+^); 4.8 of K; 5.0 of Ca; 2.0 of Mg; 1.0 of P; 1.2 of S; and (in μmol L^−1^) 28.0 of B; 54.0 of Fe; 5.5 of Mn; 2.1 of Zn; 1.1 of Cu; and 0.01 of Mo (adapted from 27) until the imposition of the drought simulation treatment. The drought treatment was performed through polyethylene glycol (PEG8000 Carbowax Sentry, Dow Chemical Comp, Midland MI, USA) addition, promoting reduction in the osmotic potential until −0.55 MPa when the plants were 51 days old. Shoot roots samples were collected two days after PEG8000 addition, 9 days (considered as severe water stress with an 80% photosynthesis reduction—data not shown), and 48 h after rehydration, when the osmotic potential was increased until −0.11 MPa [28]. A total of three biological replicates were used for each experiment. From each, plant samples were collected, immediately frozen in liquid nitrogen and stored at −80 °C (Fig. 1).

### Primer design

qPCR stability analyses were performed using eight candidate reference genes reported previously as suitable for normalizing RNA expression in sugarcane (Table 1). The sequences of reference genes actin (*ACT*), glyceraldehyde-3phosphate dehydrogenase (*GAPDH*), tubulin (*TUB*), ubiquitin (*UBQ1*/*UBQ2*), 60S ribosomal protein L35-4 (*RPL*) and 25S ribosomal RNA (*25SrRNA1*/*25SrRNA2*) were obtained from SUCEST database (http://sucest-fun.org/) (Table 1). The primers were designed using the software Primer3 [29] according to the following parameters: 58–62 °C melting temperature (Tm), 18–22 bp length, and 100–200 bp amplified fragments length. Primer pairs were tested for Tm, stability, GC content and interactions among primers using NetPrimer software (www.premierbiosoft.com/netprimer).

**Table 1:** The gene name, accession number, gene description, primer sequences and amplicon size (bp)

Gene	Accession no.	Gene description	Primer Sequence (5′–3′)	Size (bp)	References
*ACT*	CA148161	Actin	F: CTCAACCCCAAGGCTAACAG R: GGCATGAGGAAGGGCATAA	195	[30]
*GAPDH*	CA254672	Glyceraldehyde-3phosphate dehydrogenase	F: TTGGTTTCCACTGACTTCGTT R: CTGTAGCCCCACTCGTTGT	122	[30]
*TUB*	CA222437	Tubulin	F: CTCCACATTCATCGGCAACTC R: TCCTCCTCTTCTTCCTCCTCG	237	[30]
*UBQ1*	CA094944	Ubiquitin1	F: AGCCTCAGACCAGATTCCAA R: AATCGCTGTCGAACTACTTGC	110	*
*UBQ2*	CA093560	Ubiquitin2	F: CTTCTTCTGTCCCTCCGATG R: TCCAACCAAACTGCTGCTC	158	*
*RPL*	CA127053	60S ribosomal protein L35-4	F: CTGAAGACGGAGAGGGAAAA R: GGCGAAGAGAAACTAACAC	264	[31]
*25SrRNA1*	CO373883 CA171131	25S ribosomal RNA	F: ATAACCGCATCAGGTCTCCAAG R: CCTCAGAGCCAATCCTTTTCC	110	[30]
*25SrRNA2*	BQ536525	25S ribosomal RNA	F: GCAGCCAAGCGTTCATAGC R: CCTATTGGTGGGTGAACAATCC	108	[30]

* Gene sequence were retrieved from SUCEST database

### Total RNA isolation and cDNA synthesis

Total RNA was extracted from 200 mg of leaves and shoot roots tissues, according to Chang et al. [32]. Genomic DNA was removed using DNase I, following the manufacturer’s instructions (Promega, Fitchburg WI, USA). RNA concentration was determined using a spectrophotometer NanoDrop 2000 (Thermo Fisher Scientific, Wilmington DE, USA), and RNA integrity was checked in 1.0% agarose gel electrophoresis stained with ethidium bromide (1 µg mL^−1^). Reverse transcription reaction was synthesized from 1 μg of total RNA using the QuantiTect^®^ Reverse Transcription Kit following the manufacturer’s instructions (Qiagen, Foster City CA, USA).

### Quantitative PCR conditions

qPCR reactions were performed on the Applied Biosystems StepOnePlus System (Foster City CA, USA). The qPCR reactions were optimized by determining the optimal primer concentrations (0.2; 0.4; 0.8 μM) based on primer efficiencies. Briefly, a 10 μL reaction mixture consisted of 5 μL SYBR Green Super Mix (Applied Biosystems, Foster City CA, USA), 3 μL of diluted cDNA (1:30) with three primers concentration, besides a negative control (without cDNA) included for each primer combinations. The reaction thermal profile was set with an initial temperature of 95 °C for 20 s, followed by 40 cycles of 95 °C for 3 s, 60 °C for 30 s. After 40 cycles, the specificity of the amplicons was analyzed through the dissociation curve profiles (melting curve). All reactions were performed in three technical replicates in one biological replicate.

### Selection of reference genes

A set of five-fold dilutions (1:10; 1:20; 1:40; 1:80; 1:160) of cDNA from ‘IACSP94-2094’ and ‘IACSP97-7065’ were used to create the standard curves; thus the PCR efficiency (E) and correlation coefficient (*R* ^2^) were determined for each gene using the linear regression model. The PCR efficiency was estimated as E = (10^−1/slope^) − 1, with E values being confirmed by LinReg PCR 7.5 [33]. Determination of the best reference gene or best gene pair was performed using two free algorithms: NormFinder [11] and RefFinder WEB-based software [16]. The two algorithms were used to evaluate the reference gene stability looking for those genes with better stability index scores in samples of well-watered (control) and drought-stressed plants.

## Results

### qPCR of candidate genes

The primers efficiency and specificity of a set of candidate reference genes for qPCR analysis were evaluated in this study. The best primers concentration (0.2, 0.4 and 0.8 μM) in the qPCR reactions were optimized in leaves sampled in both field and greenhouse experiments, and the results here obtained according to qPCR efficiency were extrapolated for roots samples from plants growing in hydroponic solution and subjected to water deficit by adding PEG8000. Gene names, accession numbers, gene descriptions, primer sequences and efficiency, amplicon size, and correlation coefficients are listed in Table 1. *ACT*, *GAPDH* and *RPL* showed the highest efficiency at 0.8 μM for samples from both field and greenhouse, while the best primers concentration for all other candidate genes was 0.2 μM (Table 2). Complementary, primers specificity was also evaluated by dissociation step (melting curve). For each pair of primers, the melting curve showed a unique peak of fluorescence, indicating that a single fragment was amplified during qPCR for samples of leaves and shoot roots (Additional file 1: Figure S1, Additional file 2: Figure S2).

**Table 2:** Primers efficiency of the candidate reference genes

Gene	(µM)	Leaves					Shoot roots				
		*E* (%)*	*R* ^2^*	Mean *Ct*	SD	*CV* (%)	*E* (%)*	*R* ^2^*	Mean *Ct*	SD	*CV* (%)
*ACT*	0.8	93.4	0.9983	25.64	0.87	3.40	96.8	0.9973	25.89	1.21	4.68
*GAPDH*	0.8	98.7	0.9996	17.96	0.67	3.76	103.5	0.9994	22.74	0.92	4.05
*TUB*	0.2	92.2	0.9998	23.80	0.81	3.38	N.A.	N.A.	N.A.	N.A.	N.A.
*UBQ1*	0.2	104.4	0.9957	28.44	0.92	3.24	95.4	0.9966	27.61	0.86	3.13
*UBQ2*	0.2	95.7	0.9977	19.20	0.80	4.19	103.2	0.9989	21.90	1.44	6.57
*RPL*	0.8	98.7	0.9997	25.64	0.87	3.40	99.2	0.9971	21.51	1.15	5.36
*25SrRNA1*	0.2	93.1	0.9998	10.74	1.07	10.00	N.A.	N.A.	N.A.	N.A.	N.A.
*25SrRNA2*	0.2	114	0.9991	11.68	0.93	7.92	N.A.	N.A.	N.A.	N.A.	N.A.

Primer concentration (µM), standard deviation (SD), co-variance (*CV*), amplification efficiency (*E*) and correlation coefficient (*R* ^2^)

* qPCR efficiency (*E* = 10^(−1/slope^) − 1) and correlation coefficient (*R* ^2^) were determined by standard curve by excel data. N.A. means data not analyzed

The amplification efficiency (*E*) refers to the efficiency of the reaction and *E*-value of 100% means that target cDNA is duplicated in each PCR cycle of the exponential phase [34]. The correlation coefficients (*R* ^2^) refer to the matching degree of the plotted data points to the standard curve in the PCR [35]. Analysis conducted with standard curves were based on a set of fivefold dilutions of cDNA pool. Primers efficiency was confirmed by LinReg PCR 7.5 [33], and the results corroborated those from standard curve (data not shown). According to Taylor et al. [36], a good linear performance is observed when *R* ^2^ > 0.99 and primers efficiency between 90 and 110%. In leaf samples, the qPCR amplification efficiency (*E*) ranged from 92.2 to 114%, with correlation coefficients (*R* ^2^) ranging from 0.9957 to 0.9998. The mean *Ct*-values of eight candidate genes in leaf samples varying from 10.74 to 28.44 (Table 2). The co-variance (*CV*) values ranged from 3.24 to 10%. On the basis of mean *Ct*, the *25SrRNA1* had the highest expression level among the eight genes with the lowest mean *Ct*-value (10.74), and was the least stable gene with a *CV*-value of 10%. On the other hand, *UBQ1* showed the lowest expression with *Ct*-value (28.44) and also the lowest variability with a *CV*-value of 3.24% among the eight candidate reference genes. Altogether, the ranking of gene expression level by *Ct*-values was *25SrRNA1* > *25SrRNA2* > *GAPDH* > *UBQ2* > *TUB* > *ACT* > *RPL* > *UBQ1*. According to the *CV*-values, the whole rank of gene stability was *ACT* > *GAPDH* > *UBQ1* > *TUB* > *RPL* > *UBQ2* > *25SrRNA2* > *25SrRNA1*.

In shoot roots samples, qPCR amplification efficiency (*E*) ranged from 95.4 to 103.5%, with correlation coefficients (*R* ^*2*^) ranging from 0.9971 to 0.9994 (Table 2). The Ct analysis showed mean *Ct*-values of five candidate genes varying from 21.51 to 27.61. The *CV* values ranged from 3.13 to 6.57%. RPL had the highest expression level among the eight genes with the lowest mean *Ct*-value (21.51), while *UBQ1* showed the lowest expression with the highest mean *Ct*-value (27.61). Furthermore, the *UBQ1* had the lowest variability with a *CV*-value of 3.13%, while *UBQ2* was the least stable gene with a *CV*-value of 6.57%. Altogether, the ranking of gene expression level by *Ct*-values was *UBQ2* > *UBQ1* > *RPL* > *GAPDH* > *ACT*. According to the *CV*-values, the whole rank of gene stability was *UBQ1* > *GAPDH* > *ACT* > *RPL* > *UBQ2*.

### Expression stability of candidate reference genes

**Fig. 2:**
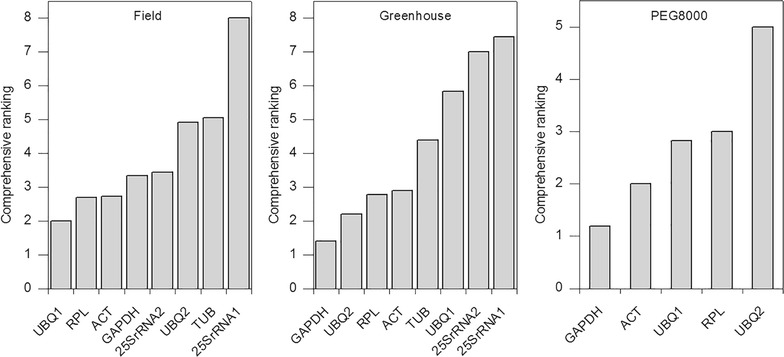
Comprehensive ranking of candidate reference genes in sugarcane genotypes subjected to drought stress. The stability of reference gene expression was measured using the *Geomean* method of RefFinder algorithm. A lower *Geomean* value denotes more stable expression

A total of eight candidate reference genes were evaluated in leaf samples of ‘IACSP94-2094’ and ‘IACSP97-7065’ genotypes under drought stress (Table 1). Samples of each experiment were analyzed individually using NormFinder and RefFinder algorithms (Table 3). For field conditions, all algorithms, except geNorm and Bestkeeper both obtained from RefFinder, identified *UBQ1* as the most stable gene (Table 3). According to RefFinder, the comprehensive ranking from the most stable to the least stable gene was: *UBQ1* < *RPL* < *ACT* < *GAPDH* < *25SrRNA2* < *UBQ2* < *TUB* < *25SrRNA1* (Fig. 2a). For greenhouse condition, all algorithms, except geNorm showed by RefFinder, indicated GAPDH as the most stable gene (Table 3). According to RefFinder, the comprehensive ranking from the most to the least stable was: *GAPDH* < *UBQ2* < *RPL* < *ACT* < *TUB* < *UBQ1* < *25SrRNA2* < *25SrRNA1* (Fig. 2b). NormFinder algorithm indicated the UBQ1 for field conditions and *GAPDH* for greenhouse as the most stable genes, which suggests that both algorithms are reliable free softwares to be used for reference gene validation. The *Geomean* method of RefFinder showed that *25SrRNA1* gene exhibited was considered as the most variable gene in leaf tissues (Table 3). NormFinder algorithm suggested *UBQ1*/*ACT* (0.164) and *25SrRNA1*/*UBQ2* (0.211) as the best pairs of genes for field and greenhouse conditions, respectively, whereas geNorm obtained from RefFinder indicated *ACT*/*RPL* for both conditions (Table 3).

**Table 3:** Analyses of reference genes evaluated according to NormFinder and RefFinder algorithms

Experimental condition	NormFinder		RefFinder							
			NormFinder		geNorm		BestKeeper		DeltaCt	
	Gene	Stability	Gene	Stability	Gene	Stability	Gene	Stability	Gene	Stability
Field	*UBQ1*	0.271	*UBQ1*	0.365	*ACT/RPL*	0	*GAPDH*	0.539	*UBQ1*	0.770
	*ACT*	0.350	*25SrRNA2*	0.494	*UBQ2*	0.559	*ACT*	0.591	*25SrRNA2*	0.820
	*25SrRNA2*	0.351	*TUB*	0.620	*UBQ1*	0.713	*RPL*	0.591	*RPL*	0.860
	*RPL*	0.358	*UBQ2*	0.629	*GAPDH*	0.759	*UBQ1*	0.617	*ACT*	0.860
	*UBQ2*	0.367	*GAPDH*	0.640	*TUB*	0.769	*25SrRNA2*	0.630	*GAPDH*	0.880
	*TUB*	0.404	*RPL*	0.663	*25SrRNA2*	0.800	*TUB*	0.639	*TUB*	0.880
	*GAPDH*	0.418	*ACT*	0.663	*25SrRNA1*	0.892	*UBQ2*	0.663	*UBQ2*	0.880
	*25SrRNA1*	0.525	*25SrRNA1*	1.054			*25SrRNA1*	0.930	*25SrRNA1*	1.170
Best pair	*UBQ1/ACT*	0.164								
Greenhouse	*GAPDH*	0.304	*GAPDH*	0.424	*ACT/RPL*	0	*GAPDH*	0.438	*GAPDH*	0.840
	*UBQ2*	0.335	*UBQ2*	0.565	*UBQ2*	0.419	*UBQ2*	0.525	*UBQ2*	0.890
	*ACT*	0.344	*TUB*	0.673	*GAPDH*	0.577	*RPL*	0.543	*ACT*	0.920
	*RPL*	0.355	*UBQ1*	0.739	*TUB*	0.729	*ACT*	0.543	*RPL*	0.920
	*TUB*	0.419	*RPL*	0.741	*UBQ1*	0.824	*TUB*	0.607	*TUB*	0.970
	*UBQ1*	0.439	*ACT*	0.741	*25SrRNA2*	0.920	*25SrRNA1*	0.630	*UBQ1*	1.010
	*25SrRNA1*	0.462	*25SrRNA2*	0.831	*25SrRNA1*	0.957	*25SrRNA2*	0.637	*25SrRNA2*	1.030
	*25SrRNA2*	0.507	*25SrRNA1*	0.902			*UBQ1*	0.675	*25SrRNA1*	1.070
Best pair	*25SrRNA1/UBQ2*	0.211								
Hydropony—PEG8000	*GAPDH*	0.150	*GAPDH*	0.239	*GAPDH/ACT*	0.477	*UBQ1*	0.750	*GAPDH*	0.65
	*ACT*	0.292	*ACT*	0.572	*RPL*	0.609	*GAPDH*	0.792	*ACT*	0.82
	*RPL*	0.362	*RPL*	0.716	*UBQ1*	0.774	*RPL*	0.848	*RPL*	0.91
	*UBQ1*	0.407	*UBQ1*	0.729	*UBQ2*	0.859	*ACT*	1.018	*UBQ1*	0.93
	*UBQ2*	0.466	*UBQ2*	0.816			*UBQ2*	1.183	*UBQ2*	0.99
Best pair	*UBQ1/ACT*	0.216

PEG8000 was used to induce water deficit in ‘IACSP94-2094’ and ‘IACSP97-7065’ genotypes. This osmolyte has a high molecular weight, decreasing the osmotic potential of nutritive solution and consequently the water availability to plants [36, 37]. Five reference genes were used to evaluate gene stability of shoot root sample hydroponic experiment (Table 1). All algorithms, except BestKeeper, indicated GAPDH gene as exhibiting the lowest expression variation, and *UBQ2* gene as the highest expression variation (Table 3). According to RefFinder, the comprehensive ranking from the most to the least stable gene was: *GAPDH* < *ACT* < *UBQ1* < *RPL* < *UBQ2* (Fig. 2c). NormFinder algorithm frequently suggested UBQ1/ACT as a best pair of primer, whereas geNorm obtained from RefFinder indicated *GAPDH*/*ACT* as a best combination (Table 3).

## Discussion

Drought is the major abiotic stress that impairs sugarcane cultivation, causing yield losses, and consequently reduction of sucrose content for sugar and ethanol production [2–4]. In order to understand the molecular basis involved in the response to abiotic stimulus such as drought, studies with qPCR have been widely conduced for characterizing gene expression patterns [8, 39]. Although qPCR is a fast, reliable and sensitive technique, normalization procedures, using suitable reference genes, are necessary to minimize variation in sample preparation and reactions [40]. In theory, a good reference gene corresponds to that one expressed constantly with a minimal change of expression, independent of experimental condition [17]. However, some studies have revealed that the expression of reference genes can undergo stability changes under abiotic stress [40].

Herein, the stability in gene expression was evaluated in two sugarcane genotypes under three experimental conditions: eight genes in leaves (field and greenhouse), and five genes in shoot roots (hydroponic solution). The analyses were conducted using NormFinder [11] and RefFinder [16] statistical algorithms, aiming to identify the best choice of single and/or pair of reference genes (Table 2). NormFinder algorithm use ANOVA for analyses of inter and intra-groups variations among samples to determine the stability value [11]. On the other hand, RefFinder integrates the available major computational algorithms (geNorm, NormFinder, Bestkeeper and delta Ct method), assigns an appropriate weight to an individual gene and calculates the geometric mean of their weights for the final ranking, named comprehensive ranking [16]. However, stability ranking of candidate reference genes can vary according to algorithms, as observed herein (Table 2).

The evaluations with NormFinder and RefFinder algorithms indicated *GAPDH* gene as the best reference genes for shoot roots samples (Table 2). When considering leaf samples, the results commonly indicated *UBQ1* and *GAPDH* genes as the most stable using both algorithms (Table 2). However, comparing the three experimental conditions in both algorithms, the results indicate differences in choosing a suitable gene due stability variations of gene expression, as noticed in other studies [13, 14, 41]. These stability variations could be associated with samples, which included different developmental stage and also different water deficit conditions [13, 14, 40]. In addition, *ACT* was the gene identified here as the most frequent when considering gene pair, as indicated by NormFinder and geNorm for all three experimental conditions.

Other authors evaluated the stability of candidate reference genes in sugarcane under drought [22–24]. Ling et al. [23] evaluated the stability of reference genes in different genotypes and tissues under abiotic stress and hormonal treatment, suggesting *GAPDH*, *eEF*-*1α* (eukaryotic elongation factor 1α) and *eIF*-*4α* (eukaryotic elongation factor 4α) genes as the most stable. Silva et al. [24] concluded that the genes *α*-*TUB* (alpha-tubulin), *H1* (histone H1) and *GAPDH* were considered the most stable reference gene in sugarcane roots under drought. In addition, Guo et al. [22] showed that *eFE*-*1α* and *GAPDH* were the most stable genes in stem of sugarcane genotypes exposed to PEG8000 and NaCl. These results taken together showed *GAPDH* gene was frequently indicated as candidate gene in sugarcane under abiotic stress, as observed in present analyses.

The indication of *GAPDH* with other genes as suitable reference genes for studies cited above suggests that they are regulated differently in different drought conditions, thus may exhibit differential expression patterns. This differential gene expression pattern was observed in three aquaporins genes for the same condition herein evaluated, i.e., field and greenhouse conditions [25], corroborating with Nicot et al. [40]. Therefore, these results indicate that reference genes need to be validated before its use for each study, since that results obtained rarely can be extrapolated to other genotypes or experimental conditions [42].

## Conclusion

In conclusion, we have validated reference genes to undergo a qPCR study involving expression in leaves and shoot roots of sugarcane under drought stress. Despite the need to validate the best reference gene for each experimental condition, this work indicates that *GAPDH* and *UBQ1* should be considered as the most suitable candidate reference genes in studies involving sugarcane leaves and roots under varying water availability in three different water deficit conditions.

## Additional files

**Additional file 1: Figure S1.** Typical dissociation curves for better concentration of pair primer in leaves samples. Pictures were taken using the qPCR instrument’s software.

**Additional file 2: Figure S2.** Typical dissociation curves for better concentration of pair primer in shoot roots samples. Pictures were taken using the qPCR instrument’s software.
