# Supplementary figures and images for: Reference genes for normalization of qPCR assays in sugarcane plants under water deficit
Source: Plant Methods. 2017 Apr 17;13:28. doi: 10.1186/s13007-017-0178-2 (PMC5392966; doi:10.1186/s13007-017-0178-2)

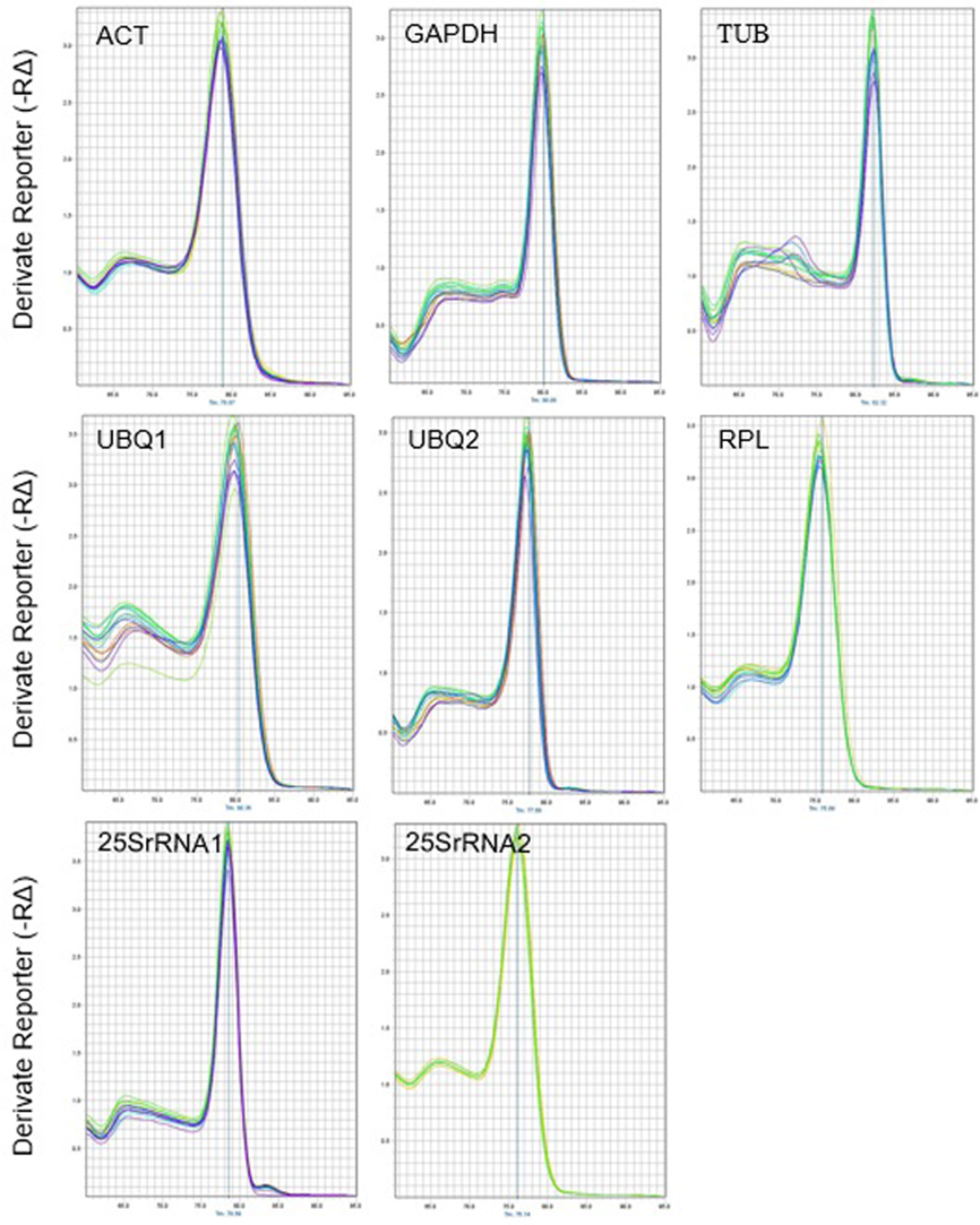

Supplement: Supplementary file 1 — Additional file 1: Figure S1. Typical dissociation curves for better concentration of pair primer in leaves samples. Pictures were taken using the qPCR instrument’s software. [file 13007_2017_178_MOESM1_ESM.tif]

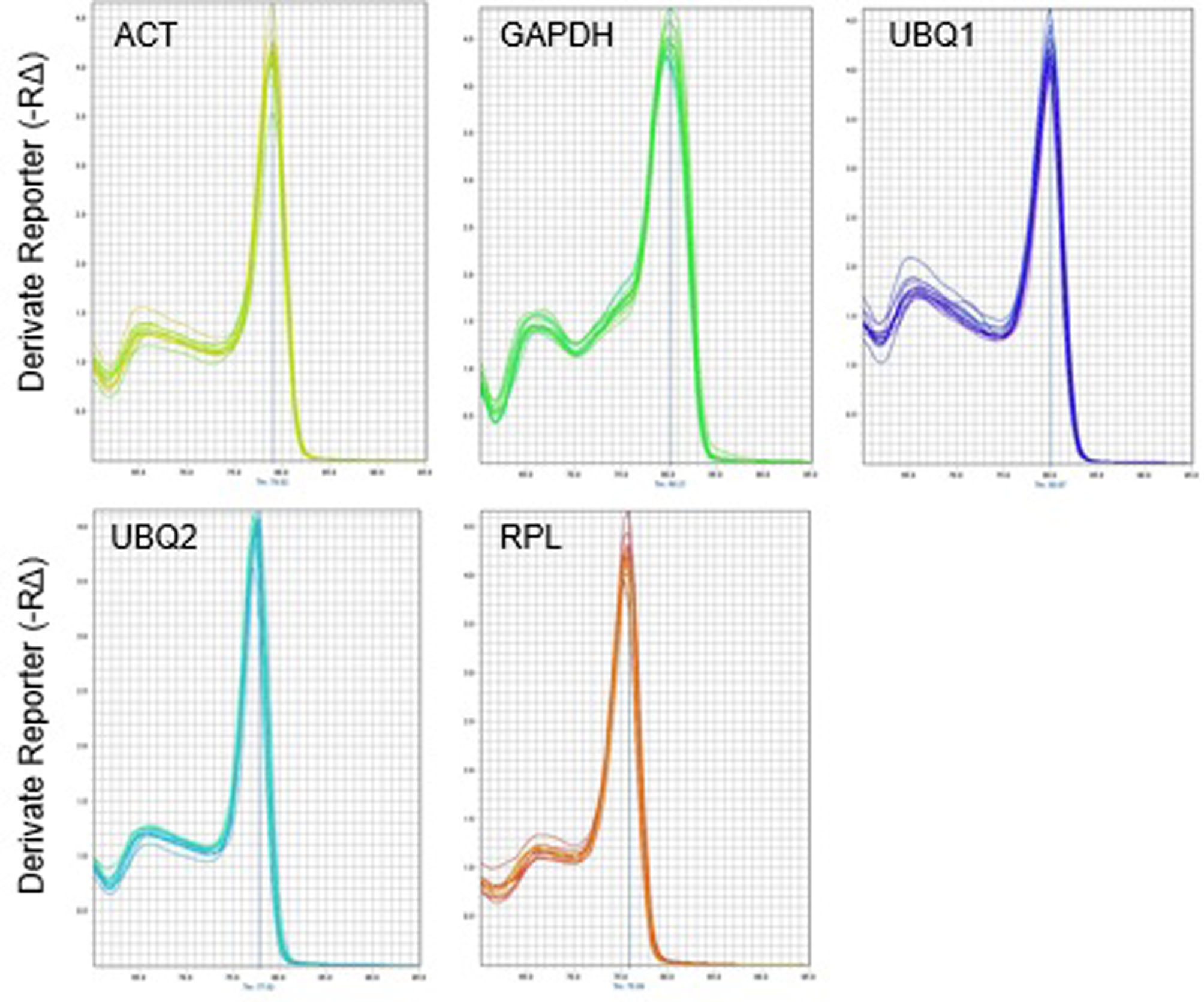

Supplement: Supplementary file 2 — Additional file 2: Figure S2. Typical dissociation curves for better concentration of pair primer in shoot roots samples. Pictures were taken using the qPCR instrument’s software. [file 13007_2017_178_MOESM2_ESM.tif]
